# Hand Musculature of the Common Marmoset (*Callithrix* *jacchus*): An Anatomical Study with Reference to the Rhesus Monkey (*Macaca mulatta*)

**DOI:** 10.3390/vetsci13030291

**Published:** 2026-03-19

**Authors:** Lise E. Collijs, Jolien Horemans, Jaco Bakker, Christophe Casteleyn

**Affiliations:** 1 Department of Morphology, Imaging, Orthopedics, Rehabilitation and Nutrition, Faculty of Veterinary Medicine, Ghent University, 9820 Merelbeke, Belgium; lise.collijs@ugent.be (L.E.C.); jolien.horemans@anicura.be (J.H.); 2 Anicura Dierenkliniek De Ark, 2235 Westmeerbeek, Belgium; 3 Animal Science Department, Biomedical Primate Research Centre, 2288GJ Rijswijk, The Netherlands; bakker@bprc.nl; 4 Department of Veterinary Sciences, Faculty of Pharmaceutical, Biomedical and Veterinary Sciences, University of Antwerp, 2610 Wilrijk, Belgium

**Keywords:** common marmoset, anatomy, myology, hand, muscles, tendons, wrist, extension, flexion

## Abstract

The common marmoset (*Callithrix jacchus*) is a New World Monkey that frequently serves as a model in biomedical research. Our knowledge of its anatomy is the fundament of the provision of the correct environment, housing, and care together with provision of accurate medical care in the case it gets wounded. In this anatomical research article, the focus is on the hand musculature, as it plays a pivotal role in the interaction with the environment and conspecifics. Surprisingly, anatomical works on the common marmoset are scarce. The available books and atlases are either outdated in terms of illustrations, providing only simplified line drawings, and/or fail to recapitulate the anatomy of the hand using contemporary anatomical nomenclature. The present work describes the myology of the hand, based on dissections of common marmoset cadavers. Color photographs taken during the subsequent steps of the examinations lead the reader through the textual descriptions. Dorsal and palmar views of the antebrachial muscles with influence on the wrist and hand as well as the intrinsic hand musculature are scrutinized. The findings are finally discussed in view of potential contradictions with the available literature. In addition, comparison is made with the rhesus monkey (*Macaca mulatta*) whose hand musculature was recently revisited. It is concluded that the hand musculature of both species is highly similar, although remarkable differences are present. This work could serve as an anatomical foundation for medical interventions of the injured hand.

## Simple Summary

The common marmoset (*Callithrix jacchus*) is often used as a primate model in scientific research. The hand of this animal is sometimes wounded during conflicts with conspecifics. Tending the wounds requires insight into the anatomy of the hand, especially the muscles and tendons. Therefore, these structures were examined in detail during the dissections of several cadavers. Color photographs were taken, and the observed muscles and tendons were described. The findings were compared with the scarce literature about the anatomy of the marmoset. Unfortunately, the literature showed outdated terms, provided only line drawings, or did not include the anatomy of the hand. The hand musculature of the common marmoset resembles that of the rhesus monkey (*Macaca mulatta*). A few dissimilarities have been observed and are discussed. This work can be used for veterinarians responsible for the medical care of wounded marmosets.

## 1. Introduction

The common marmoset or white-tufted-ear marmoset (*Callithrix jacchus*) is a New World monkey belonging to the Suborder *Haplorrhini*, Parvorder *Platyrrhini*, Family *Cebidae*, Subfamily *Callitrichinae* [1,2]. This non-human primate (NHP) is frequently exploited as a model for the human body, especially in the field of neurosciences [3]. Here, it competes with the rhesus monkey (*Macaca mulatta*) as the common marmoset presents an attractive balance between the anatomical and physiological similarities with humans on the one hand, and the small size with its associated rapid development and relatively short life span on the other hand [3]. As such, common marmosets are uniquely positioned to study the process of aging and age-related diseases [4].

Aggression is not uncommon when a new group member is introduced or when hierarchical disputes arise [5,6]. The inflicted injuries regularly result in septicemia or endotoxemia, confronting the veterinarians responsible for the medical care of the animals with the intensive monitoring and treatment of the wounded individual [7]. Injuries at the level of the extremities, such as the fingers or the tail, that fail to heal often pose the necessity of amputation [8,9]. Without any doubt, these interventions require a solid knowledge of the anatomy of the involved body parts, including the hand [10]. A well-functioning hand is pivotal to the welfare of the common marmoset as it is used for both locomotion and grasping [11].

As a consequence, a state-of-the-art, detailed anatomical atlas of the common marmoset is of the utmost importance for safeguarding the animal’s physical and mental integrity. Almost a century ago, Beattie published his opus magnum entitled “The anatomy of the common marmoset (*Hapale jacchus* Kuhl)” [12]. The use of the historical species name *Hapale jacchus* reveals that the work is out-of-date. Although the textual anatomical descriptions are much appreciated, the illustrations consisting of black-and-white line drawings often lack clarity. As far as the hand musculature is concerned, the textual descriptions are less comprehensive compared to those of the other body parts. Most significant, however, is the lack of any illustration of this body part. A recent work is the book chapter by Casteleyn and Bakker in which they describe the anatomy of the common marmoset with the biomedical researcher as the target audience [13]. This chapter focuses on the key structures, and the presented illustrations are color photographs of dissections. However, illustrations of the hand musculature are lacking, as in the work of Beattie.

The present study was initiated with the intention of portraying the gross anatomy of the muscles involved in the movements of the wrist (*carpus*) and hand (*manus*) of the common marmoset to fill the gap in the existing literature on the anatomy of this NHP. Undeniably, these muscles have an immense functional value [14,15]. The myology is systematically depicted providing the origin, course and insertion of each muscle. The textual descriptions are accompanied by multi-panel color photographs of dissected specimens. Not only the intrinsic hand musculature, but also the antebrachial musculature is scrutinized. The former includes the short muscles with origin and insertion at the level of the hand, affecting the mobility of the fingers (*digiti manus*). Antebrachial muscles have long bellies situated at the antebrachium and provoke the movements of the entire hand by influencing the wrist joint or specifically target the finger joints. The recently published manuscript on the hand musculature of the rhesus monkey served as a guide during the dissections [16]. In the discussion, comparisons will be made between the rhesus monkey and the common marmoset.

## 2. Materials and Methods

### 2.1. Animals

The frozen (−20 °C) cadavers of common marmosets (*n* = 4), two of each sex, were obtained from the biobank of the Biomedical Primate Research Centre (BPRC, Rijswijk, The Netherlands) (https://www.bprc.nl/bio-banken/bio-bank, last accessed 18 December 2025). The involved animals were euthanized for several reasons, including welfare issues and research/diagnostics. These reasons were not related to the locomotor system and, therefore, did not influence the described morphology. All animals were fasted by withholding food for 16 h. Water was never restricted. The animals were chemically immobilized by the intramuscular administration of 12 mg/kg alphaxalone (Alfaxan Multidose^®^ 10 mg/mL, Jurox Limited, London, UK). Subsequently, pentobarbital (60 mg/kg) (Euthasol^®^ 20%; AST Farma B.V., Oudewater, The Netherlands) was administered intravenously (*v. saphena parva*). It is described that this pharmacological compound in this dose does not induce tissue damage, not even at the histopathological level [17]. After they had arrived at the Faculty of Veterinary Medicine, Ghent University, Merelbeke, Belgium, they were thawed at room temperature, enabling the dissection of their thoracic limbs, in particular the antebrachium and the hand.

### 2.2. Dissection

Of each marmoset, both thoracic limbs were amputated and skinned. The antebrachium and hand were dissected layer per layer, both at the cranial respectively dorsal side, and the caudal respectively palmar side. The textual descriptions are based on the dissections of all eight limbs, but the presented photographs are from the left limbs only, as the left limb is traditionally depicted in anatomy books and atlases. Dissections were executed both by the naked eye and by means of a stereomicroscope (Olympus SZX7, Olympus Belgium, Aartselaar, Belgium). “The Anatomy of the Common Marmoset (*Hapale jacchus* Kuhl)” by Beattie [12], and the book chapter entitled “The Anatomy of the Common Marmoset” by Casteleyn and Bakker [13] were the primary sources that were consulted during the dissections. In addition, two works on the anatomy of the rhesus monkey were accessed. These included “The Anatomy of the Rhesus Monkey (*Macaca mulatta*)” by Hartman and Straus Jr. [18], and “Hand musculature of the rhesus monkey (*Macaca mulatta*): an anatomical study” by Casteleyn et al. [16]. Finally, the comparative report of primate musculature described in “Comparative Anatomy and Phylogeny of Primate Muscles and Human Evolution” by Diogo and Wood [19] was accessed.

### 2.3. Imaging

For macroscopic photographs, a Canon EOS 450D body (Canon Inc., Tokyo, Japan) combined with a Canon EF-S 18–200 mm f/3.5–5.6 IS lens (Canon Inc.) was used. Stereomicroscopic images were obtained by means of the above-mentioned stereomicroscope equipped with a charge-coupled device camera (Olympus DP50, Olympus Belgium). All photographs were taken with the specimen lying on a black background, avoiding scattering of the surrounding light. Editing of the photographs was performed using GIMP 2.10.30 (gimp.org) and Adobe Photoshop Elements 2025 (Adobe Inc., San Jose, CA, USA). This usually included cropping and equalizing the plain black background. When beneficial, adjusting the lighting and optimizing the color temperature were additionally executed. The final images were labeled using Microsoft Office PowerPoint (Microsoft, Redmond, WA, USA).

### 2.4. Anatomical Terminology

The Nomina Anatomica Veterinaria (N.A.V.) [20] and the second edition of the Terminologia Anatomica (T.A.2) [21] were consulted for the correct nomenclature of the described structures. When possible, veterinary anatomical terms are provided since the common marmoset is an NHP, which welfare and health status are supervised by the veterinarian. However, as the former reference work does not offer specific terms for the common marmoset, the human terms were borrowed from the T.A.2 [21] when a structure present in the species under investigation has no analogue in any of the domestic mammals included in the N.A.V. [20]. The Latin term is used, and written in italics, when a structure is mentioned in the text for the first time. For readability purposes, English terminology is applied to further scrutinize the structures in the textual descriptions. However, exclusively Latin terms are utilized in the figure legends.

## 3. Results

### 3.1. Dorsal View of the Antebrachial Musculature

**Figure 1:**
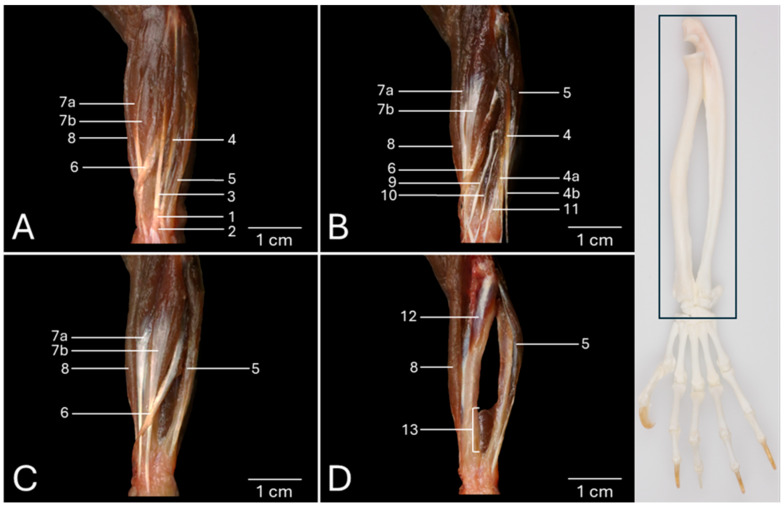
Dorsal view of the left antebrachium of the common marmoset (*Callithrix jacchus*). The black box on the skeletal figure of the left antebrachium and hand of the common marmoset indicates the location of the shown images (**A**–**D**). (**A**) Superficial layer after resection of the skin. (**B**) Deeper layer after removal of the retinaculum extensorum and the m. extensor digitorum communis. (**C**) Deeper layer after resection of the inserting tendons of the m. extensor digiti secundi/indicis proprius, the m. extensor digiti tertii proprius and the m. extensor digitorum quarti et quinti proprius. (**D**) The deepest layer after the extensor muscles have been taken away. 1: retinaculum extensorum proximale; 2: retinaculum extensorum distale; 3: m. extensor digitorum communis; 4: m. extensor digitorum quarti et quinti proprius; 4a: m. extensor digitorum quarti et quinti proprius tendon to digitus IV; 4b: m. extensor digitorum quarti et quinti proprius tendon to digitus V; 5: m. extensor carpi ulnaris; 6: m. abductor digiti primi/pollicis longus; 7a: m.extensor carpi radialis longus; 7b: m. extensor carpi radialis brevis; 8: m. brachioradialis; 9: m. extensor digiti primi/pollicis longus; 10: m. extensor digiti secundi/indicis proprius; 11: m. extensor digiti tertii proprius; 12: m. supinator; 13: m. pronator quadratus.

Figure 1A presents the dorsal view of the left antebrachium after removing the skin. On this superficial view, the double extensor retinaculum (*retinaculum extensorum proximale* (no. 1) and *retinaculum extensorum distale* (no. 2)) is visible as a reinforcement of the deep fascia immediately proximal to the wrist. It is easily recognized that the distal tendons of the extensor digitorum communis muscle (*musculus (m.) extensor digitorum communis*) (no. 3) and the extensor muscle of the fourth and fifth digits (*m. extensor digiti quarti et quinti proprius*) (no. 4), located just lateral to the former, travel through this retinaculum in the middle plane of the antebrachium. Both muscles have their origins on the lateral epicondyle of the humerus (*epicondylus lateralis*). The latter extensor muscle is laterally flanked by the extensor carpi ulnaris muscle (*m. extensor carpi ulnaris*) (no. 5), which originates on the lateral epicondyle of the humerus and the olecranon of the ulna. Its distal tendon that inserts into the base of the fifth metacarpal bone (*os metacarpale V/quintum*) is also covered by the extensor retinaculum. At the medial side of the extensor digitorum communis muscle emerges the abductor digiti primi/pollicis longus muscle (*m. abductor digiti primi/pollicis longus*) (no. 6), halfway through the antebrachium. It presents an oblique course from its origin at the proximal two thirds of the cranial side of the radius and ulna to its insertion at the abaxial side of the base of the first metacarpal bone (*os metacarpale I/primum*). The muscle belly is proximally covered by the muscle belly of the extensor digitorum communis muscle. After it has emerged from below this muscle, the muscle belly presents the transition into the distal tendon that dorsomedially crosses the distal tendons of the extensor carpi radialis longus muscle (*m. extensor carpi radialis longus*) (no. 7a) and the extensor carpi radialis brevis muscle (*m. extensor carpi radialis brevis*) (no. 7b). It additionally traverses the insertion of the brachioradialis muscle (*m. brachioradialis*) (no. 8). This muscle is located at the craniomedial side of the antebrachium. Its origin is situated in the flexion angle of the elbow, more specifically at the lateral supracondylar ridge of the humerus (*crista supracondylaris lateralis*). The inserting tendon that attaches to the distal aspect of the radius is not covered by the extensor retinaculum. Similarly, the distal tendons of both the extensor carpi radialis longus and the extensor carpi radialis brevis muscles also do not pass beneath the extensor retinaculum. The common tendon of origin of these muscles is initiated immediately lateral to the brachioradialis muscle on the lateral supracondylar ridge of the humerus. The medially located extensor carpi radialis longus muscle attaches to the base of the second metacarpal bone (*os metacarpale II/secundum*), whereas the laterally positioned extensor carpi radialis brevis muscle inserts into the base of the third metacarpal bone (*os metacarpale III/tertium*).

Both the proximal and distal extensor retinacula and the extensor digitorum communis muscle have been removed in Figure 1B. This allows for the visualization of the deeper layer of the extensor musculature of the digits that is described here from the medial to the lateral side of the antebrachium. More medially, the extensor digiti primi/pollicis longus muscle (*m. extensor digiti primi/pollicis longus*) (no. 9) can be discerned. This tiny muscle emerges distal to the muscle belly of the abductor digiti primi/pollicis longus muscle, halfway the antebrachium. Its origin can be found at the craniolateral side of the proximal aspect of the ulna. Its distal tendon crosses the inserting tendons of the extensor carpi radialis longus and the extensor carpi radialis brevis muscles before attaching to the distal phalanx (*phalanx distalis*) of the first digit, thumb or pollex (*digitus I/primus*). Lateral to this muscle lies the extensor indicis proprius muscle (*m. extensor digiti secundi/indicis proprius*) (no. 10). This small muscle originates immediately distal and lateral to the extensor digiti primi/pollicis longus muscle. The site or origin is the craniolateral side of the middle third of the ulna. Its distal tendon crosses only the inserting tendon of the extensor carpi radialis longus muscle to insert into the distal end of the proximal phalanx (*phalanx proximalis*) of the second digit or index finger (*digitus II/secundus*). More laterally and distally travels the extensor digiti tertii proprius muscle (*m. extensor digiti tertii proprius*) (no. 11) from its origin on the craniolateral side of the ulna to the distal end of the proximal phalanx of the third digit (*digitus III/tertius*). The muscle, that is located at the lateral side of the antebrachium, is the above-discussed extensor muscle of the fourth and fifth digits (no. 4). It has already been mentioned above that it originates at the lateral humeral epicondyle, lateral to the extensor digitorum communis muscle. Therefore, it is a much longer muscle compared to the extensor muscles of the first, the second and the third digits. From the single muscle belly arise two tendons in the middle section of the ulna. The medial tendon (no. 4a) inserts into the abaxial side of the proximal phalanx of the fourth digit (*digitus IV/quartus*). The lateral tendon (no. 4b) has the same insertion site, albeit at the fifth digit (*digitus V/quintus/minimus*).

All digital extensor muscles have been removed in Figure 1C. The courses of the distal tendons of the abductor digiti primi/pollicis longus, the extensor carpi radialis longus and the extensor carpi radialis brevis muscles can be examined thoroughly in this figure.

After the resection of these three muscles in Figure 1D, the supinator muscle (*m. supinator*) (no. 12) becomes evident. It was formerly obscured by the extensor carpi radialis longus muscle as it originates on the lateral humeral epicondyle. Its oblique course to the craniomedial aspect of the radius results in its effect of antebrachial endorotation. Finally, the pronator quadratus muscle (*m. pronator quadratus*) (no. 13) should be mentioned. It originates at the medial side of the distal aspect of the ulna and inserts into the lateral side of the distal aspect of the radius. The muscle fibers that run in a distomedial direction also allow for the antebrachium to endorotate.

### 3.2. Dorsal View of the Hand Musculature

**Figure 2:**
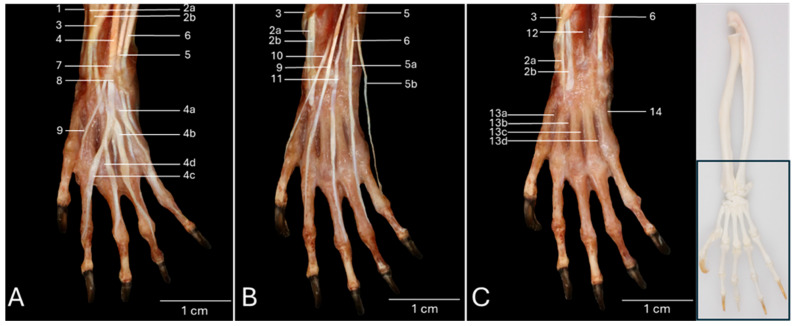
Dorsal view of the left hand of the common marmoset (*Callithrix jacchus*). The black box on the skeletal figure of the left antebrachium and hand of the common marmoset indicates the location of the shown images (**A**–**C**). (**A**): Superficial layer after resection of the skin and the fascia dorsalis manus. (**B**): Deeper layer after removal of the retinaculum extensorum and the tendons of the m. extensor digitorum communis. (**C**): Deepest layer after resection of the tendons of the m. extensor digiti primi/pollicis longus, the m. extensor digiti secundi/indicis proprius, the m. extensor digiti tertii proprius, and the m. extensor digitorum quarti et quinti proprius. 1: m. brachioradialis; 2a: m. extensor carpi radialis longus; 2b: m. extensor carpi radialis brevis; 3: m. abductor digiti primi/pollicis longus; 4: m. extensor digitorum communis; 4a: m. extensor digitorum communis tendon to digitus V; 4b: m. extensor digitorum communis tendon to digitus IV; 4c: m. extensor digitorum communis tendon to digitus II; 4d: m. extensor digitorum communis tendon to digitus III; 5: m. extensor digitorum quarti et quinti proprius; 5a: m. extensor digitorum quarti et quinti proprius tendon to digitus IV; 5b: m. extensor digitorum quarti et quinti proprius tendon to digitus V; 6: m. extensor carpi ulnaris; 7: retinaculum extensorum proximale; 8: retinaculum extensorum distale; 9: m. extensor digiti secundi/indicis proprius; 10: m. extensor digiti primi/pollicis longus; 11: m. extensor digiti tertii proprius; 12: m. pronator quadratus; 13a: m. interosseus manus dorsalis I/primus; 13b: m. interosseus manus dorsalis II/secundus; 13c: m. interosseus manus dorsalis III/tertius; 13d: m. interosseus manus dorsalis/IV; 14: m. abductor digiti quinti/minimi.

Figure 2A presents a detailed view of the left hand after removing the skin. At the level of the carpus, the following tendons can be identified from medial to lateral. First the tendon of the brachioradialis muscle (no. 1), then the tendons of the extensor carpi radialis longus (no. 2a) and brevis (no. 2b) muscles, which are dorsally crosses by the tendon of the abductor digiti primi/pollicis longus muscle (no. 3), then the common tendon of the extensor digitorum communis muscle (no. 4), then the tendon of the extensor digitorum quarti et quinti proprius muscle (no. 5), which divides into a medial tendon that runs to the fourth digit (no. 5a) and a lateral tendon to the fifth digit (no. 5b), and finally the tendon of the extensor carpi ulnaris muscle (no. 6). The proximal and distal extensor retinacula (no. 7 and no. 8, respectively) cover all these tendons, except for the tendon of the abductor digiti primi/pollicis longus muscle and the extensor carpi radialis longus and brevis muscles. Just distal to the distal extensor retinaculum, the *m. extensor digiti secundi/indicis proprius* (no. 9), extending toward the second digit, becomes visible, together with the four tendons of the extensor digitorum communis muscle that runs towards digits II to V. The first individual tendon to branch off of the common tendon of the extensor digitorum communis muscle is the tendon for the fifth digit (no. 4a). This occurs at the level of the base of the fourth metacarpal bone. The result is a common tendon for the three remaining digits. The tendon for the fourth digit (no. 4b) subsequently leaves this common trunk, giving form to the common tendon for digits II and III. Halfway the third metacarpal bone, this common tendon presents a dichotomy from which the individual tendons for the second digit (no. 4c) and the third digit (no. 4d) arise. All these individual tendons insert dorsally into the bases of the distal phalanges of the respective digits.

The tendons of the extensor digitorum communis muscle have been removed in Figure 2B. This allows for the better visualization of the tendons of the extensor digit primi/pollicis longus muscle (no. 10), the extensor digiti secundi/indicis proprius muscle, the extensor digiti tertii proprius muscle (no. 11), and the m. extensor digitorum quarti et quinti proprius muscle. This enumeration mentions the tendons from medial to lateral.

All these tendons have been resected in Figure 2C. The tendons of the extensor carpi radialis longus and brevis muscles, which are traversed in a distomedial direction by the tendon of the abductor digiti primi/pollicis longus muscle, remain at the medial side, while the tendon of the extensor carpi ulnaris muscle is fully exposed at the lateral side. In between these tendons, and more specifically in between the radius and ulna, can the pronator quadratus muscle (no. 12) be discerned. The four dorsal interosseous muscles of the hand (*mm. interossei manus dorsales*) can now be scrutinized in the four intermetacarpal spaces (*spatia interossea metacarpi*). They can be observed from the dorsal side of the hand, hence their names, but are located deep in the palm of the hand. For the description, it is important to note that the axis through the hand runs between the third and the fourth digit. The first dorsal interosseous muscle (*m. interosseus manus dorsalis I/primus*) (no. 13a) originates both at the axial side of the first metacarpal bone and the abaxial side of the second metacarpal bone to insert into the abaxial side of the proximal phalanx of the second digit. The second dorsal interosseous muscle (*m. interosseus manus dorsalis II/secundus*) (no. 13b) has its origin both at the axial side of the second metacarpal bone and the abaxial side of the third metacarpal bone to find its insertion at the abaxial side of the proximal phalanx of the third digit. The third dorsal interosseous muscle (*m. interosseus manus dorsalis III/tertius*) (no. 13c) begins at the axial sides of the third metacarpal bone and fourth metacarpal bone (*os metacarpale IV/quartum*). The attachment site is the axial side of the proximal phalanx of the third digit. Thus, the proximal phalanx of the third digits receives two tendons, i.e., the tendon from the second dorsal interosseous muscle at its abaxial side and the tendon of the third dorsal interosseous muscle at its abaxial side. Finally, the fourth dorsal interosseous muscle (*m. interosseus manus dorsalis IV*) (no. 13d) starts at the abaxial side of the fourth and the axial side of the fifth metacarpal bone. Its insertion can be found at the abaxial side of the proximal phalanx of the fourth digit. The abductor digiti minimi muscle (*m. abductor digiti quinti/minimi*) (no. 14) that is visible at the abaxial side of the fifth metacarpal bone will be elaborated below.

### 3.3. Palmar View of the Antebrachial Musculature

**Figure 3:**
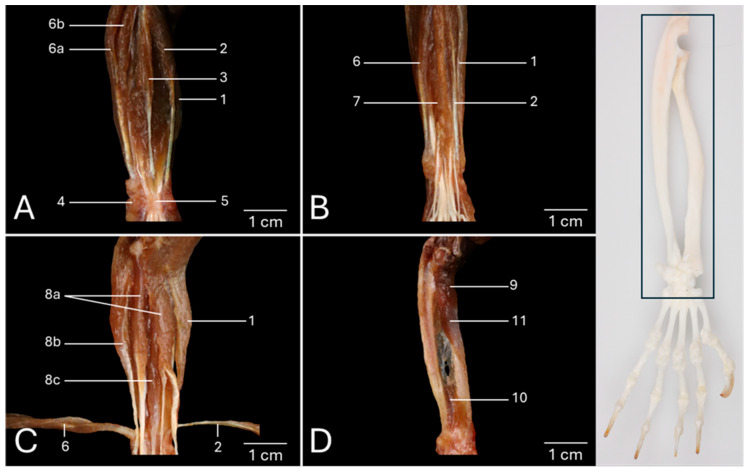
Palmar view of the left antebrachium of the common marmoset (*Callithrix jacchus*). The black box on the skeletal figure of the left antebrachium and hand of the common marmoset indicates the location of the shown images (**A**–**D**). (**A**): Superficial layer after the removal of the skin. (**B**): Superficial layer with resection of the m. palmaris longus and the retinaculum flexorum. (**C**): Deeper layer in which the superficial digital flexor muscle has been removed, and the flexor carpi ulnaris and flexor carpi radialis muscles have been retracted. (**D**): Deepest layer after resection of the m. flexor digitorum profundus, m. flexor carpi ulnaris, and m. flexor carpi radialis.1: m. brachioradialis; 2: m. flexor carpi radialis; 3: m. palmaris longus; 4: m. palmaris brevis; 5: retinaculum flexorum; 6: m. flexor carpi ulnaris caput radiale; 6b: m. flexor carpi ulnaris caput ulnare; 7: m. flexor digitorum superficialis/sublimis; 8a: m. flexor digitorum profundus caput humerale; 8b: m. flexor digitorum profundus caput ulnare; 8c: m. flexor digitorum profundus caput radiale; 9: m. supinator; 10: m. pronator quadratus; 11: m. pronator teres.

A palmar view of the left antebrachium, of which the skin has been removed, is presented in Figure 3A. The above-discussed brachioradialis muscle (no. 1) can be recognized at the medial side of the antebrachium. Immediately lateral to this muscle, but still at the medial side of the antebrachium, lies the flexor carpi radialis muscle (*m. flexor carpi radialis*) (no. 2). It runs from the medial humeral epicondyle (*epicondylus medialis*) to the base of the fifth metacarpal bone. The palmaris longus muscle (*m. palmaris longus*) (no. 3) is positioned in the median plane. The medial epicondyle of the humerus is also the site of origin of this muscle. Its long tendon fans out at the level of the metacarpus into the palmar aponeurosis (*aponeurosis palmaris*). The palmaris brevis muscle (*m. palmaris brevis*) (no. 4) can be seen at the lateral side of the flexor retinaculum. In fact, this site is where this muscle originates. It travels a short distance in a lateral direction to insert into the fascia that overlays the head of the fifth metacarpal bone. The flexor retinaculum (*retinaculum flexorum*) (no. 5), which is a reinforcement of the deep fascia at the level of the carpus, retains the tendons of the flexor digitorum superficialis muscle and the flexor digitorum profundus muscle close to this joint when it is flexed. The tendon of the flexor carpi radialis also runs within a separate canal in the retinaculum. Finally, the flexor carpi ulnaris muscle (*m. flexor carpi ulnaris*) (no. 6) can be identified at the lateral side of the antebrachium. Its radial head (*caput radiale*) (no. 6a) originates from the medial humeral epicondyle, whereas its ulnar head (*caput ulnare*) (no. 6b) has its origin on the olecranon. Halfway through the antebrachium, both muscle bellies fuse onto a single tendon that attaches to the proximal aspect of the pisiform bone (*os carpi accessorium/os pisiforme*).

The flexor retinaculum and the palmaris longus muscle have been resected in Figure 3B, shedding light on the flexor digitorum superficialis muscle (*m. flexor digitorum superficialis/sublimis*) (no. 7).

**Figure 4:**
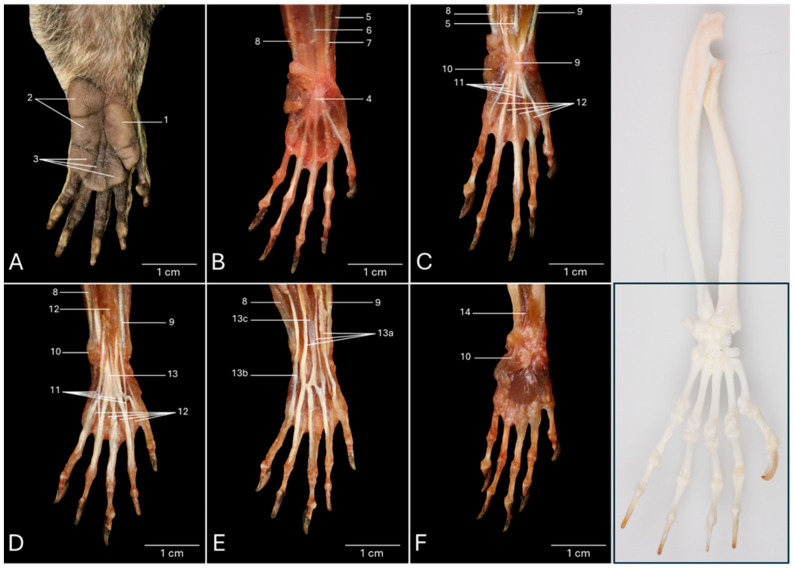
Palmar view of the left hand of the common marmoset (*Callithrix jacchus*). The black box on the skeletal figure of the left antebrachium and hand of the common marmoset indicates the location of the shown images (**A**–**F**). (**A**): Intact palm of the hand with the palmar pads. (**B**): Superficial layer after the removal of the skin. (**C**): Superficial layer after the resection of the aponeurosis palmaris. (**D**): Deeper layer after the resection of the retinaculum flexorum, the m. palmaris longus, and the mm. lumbricales manus. (**E**): Deeper layer after the resection of the m. flexor digitorum superficialis/sublimis. (**F**): Deepest layer after the m. flexor carpi ulnaris, the m. flexor carpi radialis, and the m. flexor digitorum profundus had been taken away. 1: torus thenaris; 2: torus hypothenaris; 3: tori metacarpophalangei; 4: aponeurosis palmaris; 5: m. brachioradialis; 6: m. palmaris longus; 7: flexor carpi radialis; 8: m. flexor carpi ulnaris; 9: retinaculum flexorum; 10: m. palmaris brevis; 11: m. flexor digitorum superficialis/sublimis; 12: mm. lumbricales manus; 13: m. flexor digitorum profundus; 13a: m. flexor digitorum profundus caput humerale; 13b: m. flexor digitorum profundus caput ulnare; 13c: m. flexor digitorum profundus caput radiale; 14: m. pronator quadratus.

The flexor digitorum profundus muscle (*m. flexor digitorum profundus*) can be scrutinized in Figure 3C, in which the superficial digital flexor muscle has been removed, and the flexor carpi ulnaris and flexor carpi radialis muscles have been retracted. The deep digital flexor muscle (no. 8) is composed of three heads, i.e., the humeral head (*caput humerale*) (no. 8a), the ulnar head (*caput ulnare*) (no. 8b), and the radial head (*caput radiale*) (no. 8c). The humeral head consists of two muscle bellies that both start at the medial humeral epicondyle. Halfway through the antebrachium, the medial muscle belly gives off a tendon for the first digit and one for the second digit. The lateral muscle belly provides the individual tendon for digitus IV. The ulnar head arises from the proximal half of the ulna and sends a single tendon to the fifth digit. The radial head arises from the proximal two-thirds of the radius and gives origin to two tendons at the level of the metacarpus. The medial tendon fuses with the individual tendon for the second digit from the humeral head. The lateral tendon receives a branch from the lateral muscle belly of the humeral head that provides the tendon for the fourth digit. As such, the individual tendon for the third digit is formed. This can be observed below in detail in Figure 4E.

The deep digital flexor has been resected in Figure 3D. Consequently, the above-discussed supinator muscle (no. 9) and pronator quadratus muscles (no. 10) are obvious. In addition, the pronator teres muscle (*m. pronator teres*) (no. 11) can be examined. Its origin is the medial humeral epicondyle, and the attachment site is the middle third of the radius.

### 3.4. Palmar View of the Hand Musculature

Figure 4A shows a detail of the palmar side of the intact left hand of the common marmoset. The palm of the hand (*palma manus*) is devoid of any hair and characterized by epidermal dermatoglyphics. Three pads (*tori*, singular: *torus*) can be recognized. They consist of the thick epidermis, the dermis, and a subcutaneous fat cushion (*pulvinus*). Lateral to the base of the thumb sits the large, trifid thenar pad (*torus thenaris*) (no. 1) on the thenar eminence (*eminentia thenaris*). At the opposite side of the palm lies the bifid hypothenar pad (*torus hypothenaris*) (no. 2) onto the hypothenar eminence (*eminentia hypothenaris*). At the level of the metacarpophalangeal joints of digits II–V, three metacarpophalangeal pads (*tori metacarpophalangei*) (no. 3) can be identified. One is situated at the base of the second digit, another is straddling between the third and fourth metacarpophalangeal joints, and the third is positioned at the base of the fifth digit.

The palmar aponeurosis (no. 4) becomes noticeable in Figure 4B, after skinning the hand. Furthermore, the previously discussed brachioradialis (no. 5) muscle, the palmaris longus muscle (no. 6), the flexor carpi radialis muscle (no. 7), and the flexor carpi ulnaris muscle (no. 8) are also visible.

This aponeurosis is removed in Figure 4C. As a result, the flexor retinaculum (no. 9) is in view, just like the previously discussed palmaris brevis muscle (no. 10). The tendons of the superficial digital flexor (no. 11) pass beneath the flexor retinaculum. The first, second, third, and fourth lumbrical muscles of the hand (*mm. lumbricales manus I, II, III* and *IV*) (no. 12) originate at the mediopalmar sides of the individual tendons of the flexor digitorum profundus muscle of digits II, III, IV, and V, respectively. This latter muscle will be discussed below. The individual tendons of the lumbrical muscles of the second and third digits attach to the abaxial sides of their proximal phalanges, whereas the individual tendons of digits IV and V insert into the axial sides of the respective proximal phalanges.

The tendons of the superficial digital flexor muscle can be studied after the resection of the flexor retinaculum, the palmaris longus muscle, and the lumbrical muscles of the hand (Figure 4D). Just proximal to the carpus, the muscle belly of the flexor digitorum superficialis muscle, which was initiated at the medial humeral epicondyle, divides into a medial and a lateral part. The medial part splits at the level of the carpus into a tendon for the second digit and one for the third digit. The lateral part splits more distally, halfway through the fifth metacarpal bone, into two tendons, one for the fourth and one for the fifth digit. Each of the four individual tendons attaches bilaterally to the base of the middle phalanx (*phalanx media*) of its respective digit. To this purpose, the tendons terminally branch, forming the tendinous chiasma (*chiasma tendinum*). The tendons of the deeper digital flexor muscle (no. 13) can already be appreciated between the thin tendons of the superficial digital flexor muscle.

The superficial digital flexor muscle was subsequently removed (Figure 4E), allowing for the full exposure of the deep digital flexor muscle, which, as discussed earlier, consists of three heads: a humeral (13a), an ulnar (13b), and a radial head (13c). The tendons of the deep digital flexor muscle for digits II, III, and IV travel through the tendinous chiasmata formed by the tendons of the superficial digital flexor to attach to the base of their distal phalanges. In contrast, the individual tendon for the fifth digit from the deep digital flexor muscle runs parallel at the lateral side of the corresponding tendon from the superficial digital flexor muscle and leaves the tendinous chiasma aside when travelling towards the base of the distal phalanx. For digitus I, the deep digital flexor tendon from the medial muscle belly of the humeral head runs independently and attaches directly to the base of the distal phalanx without passing through any tendinous chiasma.

Finally, the flexor digitorum profundus muscle was removed together with the flexor carpi radialis muscle and the flexor carpi ulnaris muscle (Figure 4F). So, the intrinsic musculature of the hand can be observed. This topic will be elaborated further. The previously discussed pronator quadratus muscle (no. 14) can now also be observed.

### 3.5. Intrinsic Musculature of Hand: Lateral Aspect

**Figure 5:**
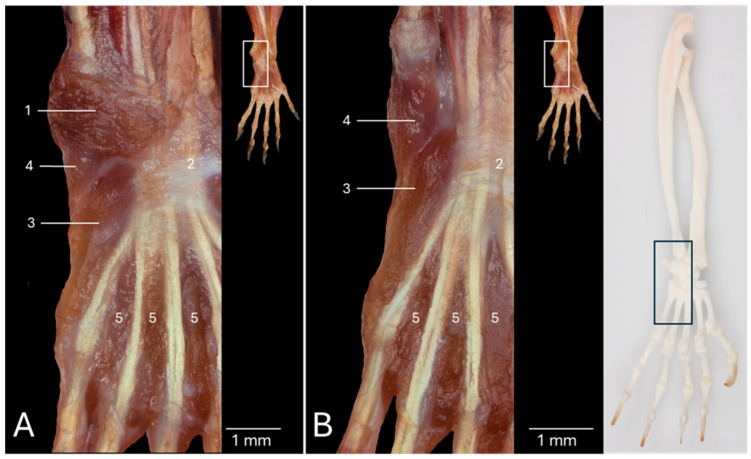
Stereomicroscopic views of the lateral aspect of the palm of the left hand of the common marmoset (*Callithrix jacchus*). The boxed area in the overview in the upper right corner shows the location of the stereomicroscopic views. The black box on the skeletal figure of the left antebrachium and hand of the common marmoset indicates the location of the shown images (**A**,**B**). (**A**): Superficial layer after the removal of the skin, the m. palmaris longus, and the aponeurosis palmaris. (**B**): Deeper layer after the resection of the m. palmaris brevis. 1: m. palmaris brevis; 2: retinaculum flexorum; 3: m. flexor digiti quinti/minimi brevis; 4: m. abductor digiti quinti/minimi; mm. lumbricales manus; 5: mm. lumbricales manus.

Figure 5A shows a stereomicroscopic view of the lateral aspect of the palm of the left hand of the common marmoset after the skin, the palmaris longus muscle, and the aponeurosis palmaris had been removed. The above-mentioned palmaris brevis muscle (no. 1) can easily be recognized. It runs from the flexor retinaculum (no. 2) and the lateral side of the palmar aponeurosis to the subcutis at the lateral side of the hand. The flexor digiti minimi brevis muscle (*m. flexor digiti quinti/minimi brevis*) (no. 3) is located mediodistal to the palmaris brevis muscle. Both muscles have the flexor retinaculum as a site of origin in common. However, the fourth carpal bone or hamate bone (*os carpale IV/quartum/os hamatum*) is the additional origin of the flexor digiti minimi brevis muscle. Its insertion can be found at the abaxial side of the proximal phalanx of the fifth digit. A glimpse of the abductor digiti quinti/minimi muscle (*m. abductor digiti quinti/minimi*) (no. 4) is visible in between both. The above-mentioned lumbrical muscles are also visible in this figure.

Figure 5B, also a stereomicroscopic view, is characterized by the removal of the palmaris brevis muscle. Consequently, the abductor digiti quinti/minimi muscle is fully exposed. It courses from the flexor retinaculum and the pisiform carpal bone towards the base of the abaxial side of the proximal phalanx of the fifth digit. The opponens digiti minimi muscle (*m. opponens digiti minimi*) will be discussed further within the chapter entitled *Intrinsic musculature of the hand: middle aspect.* The lumbrical muscles of the hand (no. 5) are additionally indicated.

### 3.6. Intrinsic Musculature of the Thumb (mm. Manus Pollicis)

**Figure 6:**
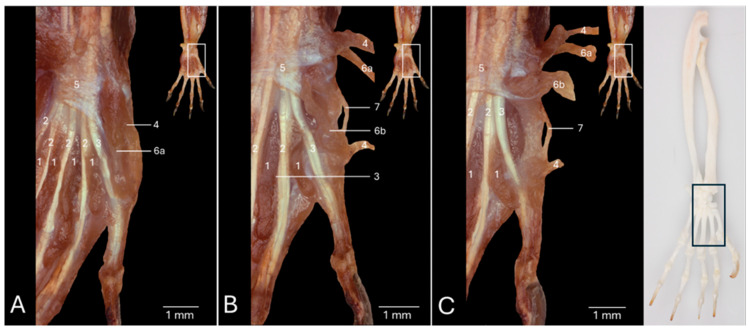
Stereomicroscopic views of the intrinsic musculature of the left thumb of the common marmoset (*Callithrix jacchus*) approached from the palmar side. The boxed area in the overview in the upper right corner shows the location of the stereomicroscopic views. The black box on the skeletal figure of the left antebrachium and hand of the common marmoset indicates the location of the shown images (**A**–**C**). (**A**): Superficial layer after removal of the skin, the m. palmaris longus, and the aponeurosis palmaris. (**B**): Deeper layer after resection of the m. abductor digiti primi/pollicis brevis muscle and the m. flexor digiti primi/pollicis brevis caput superficiale. (**C**): Deepest layer after the m. flexor digiti primi/pollis brevis caput profundum has been taken away. 1: mm. lumbricales manus I, II, III en IV; 2: m. flexor digitorum superficialis/sublimis; 3: m. flexor digitorum profundus; 4: m. abductor digiti primi/pollicis brevis; 5: flexor retinaculum; 6a: m. flexor digiti primi/pollicis brevis caput superficiale; 6b: m. flexor digiti primi/pollicis brevis caput profundum; 7: m. opponens digiti primi/pollicis.

The intrinsic musculature of the thumb that is positioned at the medial side of the hand is visualized by means of stereomicroscopic views in Figure 6. Figure 6A presents the medial aspect of the left hand of the common marmoset after the skin, the palmaris longus muscle, and the palmar aponeurosis had been removed. The lumbrical muscles of the hand (no. 1), together with the flexor digitorum superficialis muscle (no. 2) and the flexor digitorum profundus muscle (no. 3) remain intact. The abductor digiti primi/pollicis brevis muscle (*m. abductor digiti primi/pollicis brevis*) (no. 4) can be observed at the medial side of the *flexor retinaculum* (no. 5) that acts as its site of origin. The muscle originates in a distomedial direction towards the base of the abaxial side of the proximal phalanx of the thumb. The flexor digiti primi/pollicis brevis muscle (*m. flexor digiti primi/pollicis brevis*) (no. 6) sits immediately lateral to the abductor muscle of the thumb and is composed of a superficial head (*caput superficiale*) (no. 6a) and a deep head (*caput profundum*) (no. 6b). Like the abductor muscle of the thumb, the superficial head has the flexor retinaculum as origin. It also attaches to the base of the proximal phalanx of the thumb, albeit at its palmar side.

The transection of the short abductor muscle of the thumb and the superficial head of the flexor muscle of the thumb reveals the deep head of the latter muscle (Figure 6B). It is initiated at the base of the second carpal bone or trapezoid bone *(os carpale II/secundum/os trapezoideum)* and the second metacarpal bone. The axial and abaxial sides of the base of the proximal phalanx of the thumb function as the attachment sites as the tendon broadens distally. Lateral to the deep head of the flexor muscle of the thumb lies the diminutive opponens digiti primi/pollicis muscle (*m. opponens digiti primi/pollicis*) (no. 7). The medialmost aspect of the flexor retinaculum is where its origin can be found. This muscle inserts into the abaxial aspect of the body of the first metacarpal bone and does not appear to cross toward the axial side, suggesting a limited or primarily abductive rather than true oppositional function.

The deep head of the flexor digiti primi/pollicis brevis muscle is retracted in Figure 6C, allowing for the detailed inspection of the opponens digiti primi/pollicis muscle.

### 3.7. Intrinsic Musculature of the Hand: Middle Aspect

**Figure 7:**
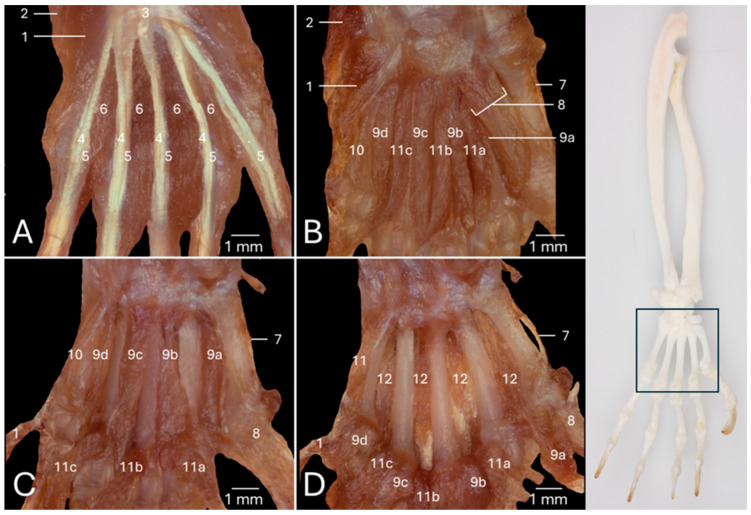
Stereomicroscopic views of the intrinsic musculature in the center of the palm of the left hand of the common marmoset (*Callithrix jacchus*). The black box on the skeletal figure of the left antebrachium and hand of the common marmoset indicates the location of the shown images (**A**–**D**). (**A**): Superficial view after the removal of the skin, the m. palmaris brevis, the m. palmaris longus, and the aponeurosis palmaris. (**B**): Deeper layer after the resection of the retinaculum flexorum, the m. flexor digitorum superficialis/sublimis, the mm. lumbricales manus, the m. flexor digitorum profundus, the m. abductor digiti primi/pollicis brevis, the m. flexor digiti primi/pollis brevis caput superficiale, and the m. flexor digiti primi/pollis brevis caput profundum. (**C**): Deeper layer after distal retraction of the m. flexor digiti quinti/minimi brevis, the mm. contrahentes digitorum manus, and the m. adductor digiti primi/pollicis. (**D**): The deepest layer after distal retraction of the mm. interossei manus palmares. 1: m. flexor digiti quinti/minimi brevis; 2: m. abductor digiti quinti/minimi; 3: flexor retinaculum; 4: m. flexor digitorum superficialis/sublimis; 5: m. flexor digitorum profundus; 6: mm. lumbricales manus; 7: m. opponens digiti primi/pollicis, 8: m. adductor digiti primi/pollicis; 9a: m. interosseus palmaris I/primus; 9b: m. interosseus palmaris II/secundus; 9c: m. interosseus palmaris III/tertius; 9d: m. interosseus palmaris IV/quartus; 10: m. opponens digiti quinti/minimi; 11a: m. contrahens digiti II/secundi; 11b: m. contrahens digiti IV/quarti; 11c: m. contrahens digiti V/quinti; 12: mm. interossei manus dorsales.

Figure 7A offers a view of the palmar side of the left hand of the common marmoset after the skin, the palmaris brevis muscle, the palmaris longus muscle and the palmar aponeurosis have been removed. As a result, the flexor digiti quinti/minimi brevis muscle (no. 1) and the abductor digiti quinti/minimi muscle (no. 2), which were elaborated above, can be observed lateral to the flexor retinaculum (no. 3). Distal to this flexor retinaculum emerge the tendons of the superficial (no. 4) and deep digital flexor muscles (no. 5). In addition, the four lumbrical muscles of the hand (no. 6) that are attached to the mediopalmar sides of the individual tendons of the deep digital flexor muscles for digits II to V are easily recognizable.

In Figure 7B, the flexor retinaculum, the tendons of the superficial and deep digital flexor muscles, the lumbrical muscles of the hand, and some of the intrinsic muscles of the thumb, including the de abductor digiti primi/pollicis brevis muscle, and the superficial and deep heads of the flexor digiti primi/pollis brevis muscle, have been resected. The above-discussed diminutive opponens digiti primi/pollicis muscle (no. 7) is consequently discernible. At the lateral side of the first metacarpal bone, the adductor digiti primi/pollicis muscle (*m. adductor digiti primi/pollicis*) (no. 8) can be seen arising from the base of the second metacarpal bone. It attaches to the axial side of the proximal phalanx of the thumb.

Figure 7B is particularly suited to examine the four palmar interosseous muscles of the hand (*mm. interossei manus palmares*) (no. 9). In contrast to the dorsal interosseous muscles of the hand, these are not paired and therefore do not arise from the two adjacent sides of neighboring metacarpal bones. The first, second, third, and fourth palmar interosseous muscles (*m. interosseus palmaris I/primus* (no. 9a)*, m. interosseus palmaris II/secundus* (no. 9b), *m. interosseus palmaris III/tertius* (no. 9c), and *m. interosseus palmaris IV/quartus* (no. 9d), respectively) arise from the axial sides of the first, second, fourth, and fifth metacarpal bones, respectively. Their insertions can be found at the axial aspects of the base of the proximal phalanx that succeeds the metacarpal bone of origin. It should be noticed that the first palmar interosseous muscle is partly covered by the adductor digiti primi/pollicis muscle, so that only its lateral portion is visible in this figure. Lateral to the fourth palmar interosseous muscle lies the opponens digiti quinti/minimi muscle (no. 10). The flexor retinaculum and the fourth carpal bone are the structures from which this muscle arises. The insertion can be found at the abaxial side of the base of the fifth metacarpal bone.

The three contrahens muscles of the hand (*mm. contrahentes digitorum manus*) (no. 11) can also be scrutinized using Figure 7B. The broad aponeurosis they share is connected to the distal aspects of the trapezoid bone and the capitate or third carpal bone (*os capitatum* or *os carpale III/tertium*), and to the bases of the second and third metacarpal bones. The contrahens muscle of the second digit (*m. contrahens digiti II/secundi*) (no. 11a), the contrahens muscle of the fourth digit (*m. contrahens digiti IV/quarti*) (no. 11b), and the contrahens muscle of the fifth digit *m. contrahens digiti V/quinti* (no. 11c) attach to the axial sides of the bases of the proximal phalanges of the digits to which their names refer.

The flexor digiti quinti/minimi brevis muscle, the contrahentes muscle of the hand, and the adductor digiti primi/pollicis muscle have been retracted in distal direction in Figure 7C. As a result, the palmar interosseous muscles of the hand can be studied in more detail. Particularly the first palmar interosseous muscle is fully exposed now.

Finally, the palmar interosseous muscles of the hand were also retracted in distal direction, allowing for the visualization of the four dorsal interosseous muscles of the hand (no. 12), which were discussed above using the dorsal approach (Figure 7D).

### 3.8. Detail Liggamenta Anularia Digitorum en Liggamenta Cruciformes Digitorum

**Figure 8:**
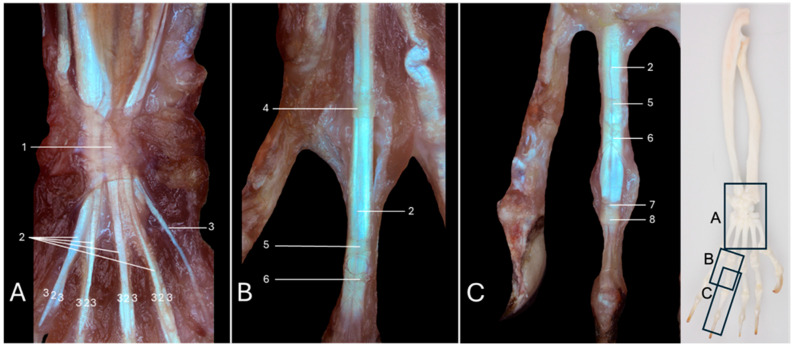
Stereomicroscopic views of the retinaculum flexorum, and of the ligamenta anularia and ligamenta cruciformes of digitus IV of the left hand of the common marmoset (*Callithrix jacchus*). The black boxes on the skeletal figure of the left antebrachium and hand of the common marmoset indicate the locations of the shown images (**A**–**C**). (**A**): Detail of the retinaculum flexorum located at the palmar side of the hand at the level of the carpal joint. (**B**): Detail of the ligamentum anulare at the level of the metacarpophalangeal joint, and of the ligamentum anulare and ligamentum cruciforme located in the middle segment of the proximal phalanx of digitus IV. (**C**): Detail of the ligamentum anulare and ligamentum cruciforme located in the middle segment of the proximal phalanx of digitus IV, and of the ligamentum anulare and ligamentum cruciforme at the level of the middle segment of the middle phalanx of digitus IV. 1: retinaculum flexorum; 2: m. flexor digitorum superficialis/sublimis; 3: m. flexor digitorum profundus; 4: lig. anulare (articulatio metacarpophalangea); 5: lig. anulare (phalanx proximalis); 6: lig. cruciforme (phalanx proximalis); 7: lig. anulare (phalanx media); 8: lig. cruciforme (phalanx media).

The stereomicroscopic view presented in Figure 8A shows a detail of the flexor retinaculum (no. 1). It retains the tendons of the superficial digital flexor muscle (no. 2) and the deep digital flexor muscle (no. 3) against the carpus when the carpal joint is flexed. The tendon of the flexor carpi radialis muscle also runs within a separate canal in the retinaculum. At the level of the digits, similar structures can be observed. The annular ligaments of the digits (*ligamenta (ligg.) anularia digitorum*) and the cruciform ligaments of the digits (*ligg. cruciformes digitorum*) compose the *vaginae fibrosae digitorum manus*, which are fibrous bands that retain the tendons of the flexor muscles against the digits when these are flexed. Each digit presents a tripartite fibrous vagina (*vagina fibrosa digiti)*, with the exception of the thumb, where it is only a single structure. Figure 8B presents a detail of the metacarpophalangeal joint of the fourth digit, showing the annular ligament (*ligamentum (lig.) anulare*) (no. 4). It can be observed that only parts with transverse fibers (*pars anularis vaginae fibrosae*) are present here. The individual tendons of the superficial and deep digital flexor muscles travel through this ligamentous ring. The longitudinal groove in the midline of the tendon of the superficial digital flexor muscle is already apparent at this level. This is the prelude to the formation of the tendinous chiasma. In addition to the transverse fibers of the annular ligaments, crossed fibers are seen immediately distal to these in the middle segments of the proximal phalanges and in the proximal segments of the middle phalanges of the second to fifth digits. These cruciform ligaments (*ligamentum (lig.) cruciforme digiti*) (no. 6) are also visible in Figure 8B,C. Both the annular and cruciform ligaments of the digits secure the positions of the tendons of the superficial and deep digital flexor muscles. The position of the distal fibrous vagina corresponds with the location of the tendinous chiasma, formed by the attachment of the split tendons of the superficial digital flexor muscle at the axial and abaxial sides of the proximal aspect of the middle phalanges. It should be noticed that the tendon of the deep digital flexor muscle to the thumb is retained by a single annular ligament that is located at the level of the metacarpophalangeal joint.

An overview of all the described muscles with their origins, insertions and mode of actions can be found in Table 1.

## 4. Discussion

The present study has elaborated on the hand musculature of the common marmoset by means of dissections performed on four cadavers. This low number of dissected specimens has enabled the investigators to identify a few anatomical variations, but the incidence of these remains to be elucidated. It was not the intention to perform functional or biomechanical analyses. Therefore, the significance of these variations and, to a larger extent, the functional meaning of specific muscular configurations that were observed in the common marmoset remains enigmatic. Future studies could link the here-presented anatomical facts to electromechanical or kinematic data, which has particular value in the field of comparative primatology and neurosciences. Where the scarce literature on the anatomy of the common marmoset fell short as a dissection guide, it was complemented by publications on the anatomy of the rhesus monkey. Although anatomical differences between these New World and Old World monkeys were expected, this approach allowed for the comparison between both primate species that are often included as models in biomedical research [19]. The present discussion will focus on the observed dissimilarities, emphasizing the need for specific anatomical work on the common marmoset. The numerous multi-panel figures showing color photographs of the antebrachial musculature with influence on the hand mobility, as well as the intrinsic hand musculature, taken from various points of view, grant the application of the present work as an anatomical atlas during wound treatment and surgery. It was chosen to apply veterinary anatomical nomenclature since the medical management of common marmosets housed in research facilities or zoos is the responsibility of veterinarians. However, the use of a uniform anatomical terminology was challenging. Given the fact that the common marmoset is an NHP, the choice for the N.A.V. as a guide in this matter was evident [20]. Unfortunately, terms for structures specific for the common marmoset and not existing in any of the domestic mammals included in the N.A.V., i.e., rabbit, cat, dog, horse, cattle, sheep, goat, and pig, had to be borrowed from the T.A.2 [21].

In the common marmoset, the extensor carpi radialis longus muscle inserts into the base of the second metacarpal bone, consistent with the condition observed in the rhesus monkey [16,18,22]. Similarly, the extensor carpi radialis brevis inserts into the base of the third metacarpal bone in both species [16,18,22]. In contrast, Beattie reported insertions into the first and second digits, respectively [12]. However, this interpretation has been contradicted by subsequent studies and is also not supported by the present findings [13].

The second muscle with dissimilarities between the common marmoset and the rhesus monkey is the extensor carpi ulnaris muscle. Almost a century ago, it was suggested that this muscle not only has a massive origin on the humerus but also has a smaller origin on the olecranon [12]. The present study failed to identify such a secondary origin on the ulna, nor did previous studies [16,18,22]. It is hypothesized that the secondary origin described by Beattie merely represents a band of fascia [12].

Further elaborating on the extensor musculature, the extensor digitorum quarti et quinti proprius muscle should be discussed as it presents a small divergence with the rhesus monkey, and a clear distinction with man. In the common marmoset, two tendons arise halfway along the ulna from its single muscle belly. The medial tendon inserts into the abaxial side of the base of the proximal phalanx of the fourth digit, enabling the extension of the fourth digit. Extension of the fifth digit is made possible by the insertion of the lateral tendon into the abaxial side of the base of the proximal phalanx of the fifth digit. The divergence in the rhesus monkey lies in the fact that the single muscle belly presents a transition into a single tendon that only divides at the level of the base of the fifth metacarpal bone. The medial tendon inserts into the abaxiodorsal side of the proximal phalanx of the fourth digit. The lateral tendon has the abaxiodorsal side of the middle phalanx of the fifth digit as its insertion [16,18,22]. It is interesting to note that this muscle is reduced in humans. As it only extends the fifth digit, it is called the extensor digiti minimi muscle (*m. extensor digiti minimi*) [23,24]. In analogy with domestic mammals, this extensor muscle that is located lateral to the extensor digitorum communis muscle can also be designated as the extensor digitorum lateralis muscle (*m. extensor digitorum lateralis*) [25].

Following the tendons of the extensor digiti primi/pollicis longus muscle and the extensor digiti secundi/indicis proprius muscle in proximal direction, it is perceived that their transitions into the muscle bellies takes place proximal to the extensor retinaculum. In addition, it is worthwhile to mention that both muscle bellies are largely merged in the common marmoset. This observation is confirmed by several authors [12,19,26,27]. In contrast, the rhesus monkey presents two clearly separated muscles. Furthermore, the tendon of the extensor digiti primi/pollicis longus muscle appears distal to the extensor retinaculum in this species [16,18,22].

The dissections revealed that the adductor digiti primi/pollicis muscle of the common marmoset consists of a single muscle belly with no distinction between an oblique and a transverse head. Beattie, however, suggests such a partition in one of his line drawings but omits to label these [12]. Nonetheless, it is highly conceivable that the dissections correctly identified the absence of the oblique and transverse heads in the common marmoset, since other authors came to the same conclusion [19,28]. In the rhesus monkey, the adductor digiti primi/pollicis muscle is undoubtedly composed of an oblique and a transverse head [16,18,22,29], which is also the case in humans [23,24,25]. As a result, the adductor digiti primi/pollicis muscle of the rhesus monkey is broad and obscures the contrahens digiti II/secundi muscle that attaches to the axial side of the base of the proximal phalanx of the second digit. In contrast, the modest adductor digiti primi/pollicis muscle of the common marmoset does not hide the contrahens muscle of the second digit.

It should be noted that, in contrast to the human hand, where three palmar interosseous muscles are described [23,24], four palmar interosseous muscles were observed in the common marmoset in the present study. This observation underscores the complex and sometimes ambiguous relationships among the palmar interosseus muscles, contrahentes muscles, and the adductor digiti primi/pollicis in non-human primates, as documented in previous studies [19,26,27,28,29,30,31].

Like the extensor musculature, the flexor musculature of the common marmoset’s hand presents some differences with that of the rhesus monkey. A minor discrepancy concerns the flexor digiti quinti/minimi brevis muscle that has its origin on both the flexor retinaculum and the hamate bone in the common marmoset. In contrast, this muscle originates only from the flexor retinaculum in the rhesus monkey [16].

Major differences can be identified when comparing the flexor digitorum profundus muscle of the common marmoset and the rhesus monkey. In the former species, this muscle is composed of a humeral, ulnar, and radial head. The humeral head is absent in the rhesus monkey [16,18,22]. The other two heads are very comparable between both NHP species as regards their positions within the antebrachium. Further complicating the composition of the deep digital flexor muscle in the common marmoset, it should be mentioned that its humeral head consists of two muscle bellies. The medially located belly sends tendons to the thumb and the index finger, while the lateral belly provides the tendon for the fourth digit. Since the flexor digiti primi/pollicis longus muscle (*m. flexor digiti primi/pollicis longus*), which is typical for humans [23,24,25], is absent in both NHP species [16,18,22], the tendon from the flexor digitorum profundus muscle to the thumb is substantial. The ulnar head is uncomplicated as it gives off a single tendon to the fifth digit. In contrast, the radial head divides into two tendons. The medial tendon fuses with the tendon of the medial belly of the humeral head for the index finger. The lateral tendon receives a tendinous branch from the lateral belly of the humeral head to form the tendon for the third digit. The situation is straightforward in the rhesus monkey as the common tendon of the flexor digitorum profundus muscle splits into five individual tendons, one for each of the five digits [16,18,22]. In contrast, the human deep digital flexor muscle presents a comparable organization to the superficial digital flexor muscle of the common marmoset, which means that no tendon is sent to the thumb. As a consequence, the flexor digiti primi/pollicis longus muscle has developed in man [23,24,25].

Controversy exists on the presence of the opponens digiti primi/pollicis muscle in the common marmoset. Since it is generally accepted that marmosets have non-opposable thumbs [30], it is expected that the opponens digiti primi/pollicis muscle is absent in the common marmoset. This view is shared by Beattie, who could not identify this muscle in any of the 14 common marmosets he dissected [12]. Based on their dissection of one specimen, Diogo and Wood state that the opponens digiti primi/pollicis muscle is not present as a distinct muscle in *Callithrix jacchus* [19]. Moreover, it does not even seem to be fused with the flexor digiti primi/pollicis brevis muscle [19]. These authors have added this statement in reply to the observation made by Jouffroy and Lessertisseur [31] that the opponens digiti primi/pollicis and the flexor digiti primi/pollicis brevis are usually fused in *Callithrix*. Nevertheless, Senft declares that the opponens digiti primi/pollicis muscle is present as a distinct muscle in the common marmoset, running from the flexor retinaculum and trapezium bone to the first metacarpal bone [32]. Dunlap and co-authors likewise report on the presence of a diminutive, rudimentary muscle in that region, which to the best of their knowledge corresponds to the opponens digiti primi/pollicis muscle [27]. The dissections performed in the framework of the present study have also revealed the presumptive opponens digiti primi/pollicis muscle sitting lateral to the deep head of the flexor muscle of the thumb. Without any doubt, the opponens digiti primi/pollicis muscle is present in the rhesus monkey [16,18,22].

Finally, species-specific variations can also be recognized when examining the opponens digiti quinti/minimi muscle. Beattie suggests that this muscle is lacking in the common marmoset [12]. Brooks [33] and Diogo and Wood [19], on the other hand, were able to identify this muscle. Their statements that it consists of a single muscle belly were confirmed by the present study. This situation contrasts with that in the rhesus monkey, in which a superficial and a deep part can be acknowledged [18,19,29].

## 5. Conclusions

The present work focused on the detailed representation of the antebrachial and hand musculature of the common marmoset. Color photographs arranged in multi-panel figures guide the reader through the dissections from the superficial plane to the deeper layers. As a result, this work can serve as a dissection guide and anatomical atlas, which could be valuable in the planning of medical interventions and in future anatomical studies, e.g., on the arthrology of the forelimb, complementing the locomotor system of the common marmoset [34]. Comparison was made with the rhesus monkey, as this NHP species is often encountered in research facilities together with the common marmoset. The hand musculature of both species is largely similar. However, a number of fundamental differences were highlighted. These have to be taken into consideration when the veterinarian responsible for the medical care of common marmosets or the biomedical researcher applying this NHP species as a model relies on their knowledge of the more extensively described rhesus monkey.

## Figures and Tables

**Table 1:** List of the muscles of the common marmoset’s hand with indication of the origin, insertion, and action, in alphabetical order.

Muscle	Origin	Insertion	Action
**m. abductor digiti primi/pollicis brevis**	Flexor retinaculum	Abaxial, base of proximal phalanx of digit I	Abduction of digit I
**m. abductor digiti primi/pollicis longus**	Proximal two-thirds of the cranial side of the radius and ulna	Abaxial, base of the 1st metacarpal bone	Abduction of digit I
**m. abductor digiti quinti/minimi**	Flexor retinaculum and pisiform carpal bone	Abaxial, base of the proximal phalanx of digit V	Abduction of digit V
**m. adductor digiti primi/pollicis**	Bases of the 2nd metacarpal bones	Axial, proximal phalanx of digit I	Flexion of digit I
**m. brachioradialis**	Lateral supracondylar ridge of the humerus	Distal aspect of the radius	Flexion of the elbow
**m. extensor carpi radialis brevis**	Lateral humeral epicondylar crest	Dorsal, base of the 3rd metacarpal bone	Extension of the wrist
**m. extensor carpi radialis longus**	Lateral humeral epicondylar crest	Dorsal, base of the 2nd metacarpal bone	Extension of the wrist
**m. extensor carpi ulnaris**	The lateral humeral epicondyle and the olecranon of the ulna	Dorsal, base of the 5th metacarpal bone	Extension of the wrist
**m. extensor digiti primi/pollicis longus**	Craniolateral side of the proximal aspect of the ulna	Dorsal, distal phalanx of digit I	Extension of digit I
**m. extensor digiti quarti et quinti proprius**	Lateral humeral epicondyle	Dorsolateral, abaxial of the proximal phalanges of digits IV and V	Extension of digits IV and V
**m. extensor digiti secundi/indicis proprius**	Craniolateral aspect of the middle of the ulna	Dorsal, distal end of the proximal phalanx of digit II	Extension of digit II
**m. extensor digiti tertii proprius**	Craniolateral aspect of the middle of the ulna	Dorsal, distal end of the proximal phalanx of digit III	Extension of digit III
**m. extensor digitorum communis**	Lateral humeral epicondyle	Dorsal, base of the distal phalanges of digits II to V	Extension of digits II to V
**m. flexor carpi ulnaris**	Medial humeral epicondyle (caput radiale) and the olecranon (caput ulnare)	Proximal aspect of the accessory (pisiform) carpal bone	Flexion of the wrist
**m. flexor digiti primi/pollicis brevis caput profundum**	Base 2nd metacarpal bone and the second carpal bone	Lateral and medial aspects of the base of the proximal phalanx of digit I	Flexion of digit I
**m. flexor digiti primi/pollicis brevis caput superficiale**	Flexor retinaculum	Palmar, base of the proximal phalanx of digit I	Flexion of digit I
**m. flexor digiti quinti/minimi brevis**	Flexor retinaculum and hamate carpal bone	Abaxial, base of the proximal phalanx of digit V	Flexion of digit V
**m. flexor digitorum profundus**	Medial humeral epicondyle (caput humerale), proximal half of the ulna (caput ulnare) and proximal two-thirds of the radius (caput radiale)	Palmar, bases of the distal phalanges of all five digits	Flexion of all five digits
**m. flexor digitorum superficialis/sublimis**	Medial humeral epicondyle	Palmar, bases of the middle phalanges of digits II to V	Flexion of digits II to V
**m. opponens digiti primi/pollicis**	Flexor retinaculum	Abaxial aspect of the body of the 1st metacarpal bone	Diminutive, abduction of digit I
**m. opponens digiti quinti/minimi**	Flexor retinaculum and the fourth carpal bone	Abaxial aspect of the base of the 5th metacarpal bone	Flexion, adduction, and medial rotation of digit V
**m. palmaris brevis**	Lateral aspect of the flexor retinaculum	Subcutis at the lateral side of the hand	Tensing the hypothenar skin towards the palm
**m. palmaris longus**	Medial humeral epicondyle	Palmar aponeurosis	Flexion of the wrist through tension on the palmar aponeurosis
**mm. contrahentes digitorum manus**	Distal aspect of the trapezoid bone and the capitatum or third carpal bone, and to the bases of the 2nd and 3rd metacarpal bones	Palmar, axial side of the base of the proximal phalanx of digits II, IV and V	Adduction of the digits
**mm. interossei manus dorsales**	Dorsal, intermetacarpal clefts I to IV	Sides of the proximal phalanges of digits II to V	Abduction of the digits
**mm. interossei manus palmares**	Palmar, intermetacarpal clefts II to IV	Axial aspects of the bases of the proximal phalanx of the first, second, fourth and fifth metacarpal bones for respectively digit I, II, IV and V.	Adduction of the digits
**mm. lumbricales manus**	Mediopalmar surfaces of the deep flexor tendons to digits II to V	Abaxial side of the proximal phalanges of digits II and III, axial side of the proximal phalanges of digits IV and V	Flexion of the fingers in the metacarpophalangeal joints

## Data Availability

The original contributions presented in this study are included in the article. Further inquiries can be directed to the corresponding author.
